# Effects of Methamphetamine on Within- and Between-Network Connectivity in Healthy Adults

**DOI:** 10.1093/texcom/tgab063

**Published:** 2021-10-29

**Authors:** Michael Malina, Sarah Keedy, Jessica Weafer, Kathryne Van Hedger, Harriet de Wit

**Affiliations:** Department of Psychiatry and Behavioral Neuroscience, University of Chicago, 5841 MarylandAvenue, Chicago, IL 60637; Department of Psychiatry and Behavioral Neuroscience, University of Chicago, 5841 S Maryland Avenue, Chicago, IL 60637; Department of Psychiatry and Behavioral Neuroscience, University of Chicago, 5841 MarylandAvenue, Chicago, IL 60637; Department of Psychiatry and Behavioral Neuroscience, University of Chicago, 5841 S Maryland Avenue, Chicago, IL 60637; Department of Psychiatry and Behavioral Neuroscience, University of Chicago, 5841 S Maryland Avenue, Chicago, IL 60637; Department of Psychology, University of Kentucky, 106-B Kastle Hall, Lexington, KY 40506; Department of Psychiatry and Behavioral Neuroscience, University of Chicago, 5841 S Maryland Avenue, Chicago, IL 60637; Department of Clinical and Neurological Sciences, University of Western Ontario, University Hospital, 339 Windermere Road, London, Ontario N6A 5A5, Canada; Department of Psychiatry and Behavioral Neuroscience, University of Chicago, 5841 MarylandAvenue, Chicago, IL 60637; Department of Psychiatry and Behavioral Neuroscience, University of Chicago, 5841 S Maryland Avenue, Chicago, IL 60637

**Keywords:** fMRI, functional connectivity, healthy humans, methamphetamine, resting state

## Abstract

Methamphetamine (MA) abuse remains an urgent public health problem. Understanding how the drug affects brain function will help to identify how it leads to abuse and dependence. Previous studies indicate that MA and other stimulants have complex effects on resting state functional connectivity. Here, we used a hypothesis-free approach to examine the acute effects of MA (20 mg oral) versus placebo on neural connectivity in healthy adults. Using networks identified by an independent component analysis with placebo data, we examined the effects of MA on connectivity within and between resting state networks. The drug did not significantly alter connectivity within networks. MA did alter connectivity between some networks: it increased connectivity between both the thalamus and cerebellum to sensorimotor and middle temporal gyrus. However, MA decreased connectivity between sensorimotor and middle temporal gyrus networks. MA produced its expected subjective effects, but these were not significantly related to connectivity. The findings extend our knowledge of how MA affects connectivity, by reporting that it affects between-network connectivity but not within-network connectivity. Future studies with other behavioral measures may reveal relationships between the neural and behavioral actions of the drug.

## Introduction

Methamphetamine (MA) is a prototypic stimulant drug with both therapeutic value and potential for misuse. Although much is known about its receptor actions and behavioral effects in both laboratory animals and humans, less is known about the acute effects of MA on brain function in humans. Using a range of different techniques, imaging studies can provide new insights into the actions of stimulant drugs on brain function and improve our understanding of how the drugs produce cognitive, mood-altering, and abuse-related effects (Abi-Dargham et al. 2003; Martinez et al. 2007). Functional magnetic resonance imaging (MRI), for example, has been used to study neural function after chronic use of stimulants (Li et al. 2020), as a predictor of treatment responses to stimulants in individuals with attention deficit (ADHD) (Norman et al. 2021), and to study acute effects of stimulants in healthy volunteers (Jiang et al. 2021). Pharmacological MRI has been used to investigate regional neural activation during presentation of tasks and/or elicitation of memories (Knutson et al. 2004; Carhart-Harris et al. 2014), as well as connectivity among brain regions, either in the resting state or during performance of tasks (Carhart-Harris. 2016). Acute drug challenge studies of functional connectivity (FC), such as the study presented here, can shed light on the neural mechanisms underlying both therapeutic and unwanted effects of these drugs.

Several studies have examined effects of stimulant drugs on FC in the resting state in healthy adults (Ramaekers et al. 2013; Mueller et al. 2014; Konova et al. 2015; Schrantee et al. 2016; Gorka et al. 2020; Weafer et al. 2020). For example, Schrantee et al. examined effects of intravenous d-amphetamine on selected resting state networks (RSNs) deemed to be of interest and defined with an independent component analysis (ICA). Amphetamine decreased FC of the anterior cingulate within the cortico-striatal-thalamic network and within the anterior default mode network. The drug also decreased connectivity among parietal and temporal cortical nodes within posterior default mode and salience executive networks, but did not alter frontoparietal networks. The authors examined drug-induced changes in connectivity in relation to changes in dopamine function assessed with single photon emission computed tomography (SPECT) and found that decreases in striatal system connectivity were positively correlated with increases in dopamine release. Using a seed-based connectivity approach, Weafer et al. (2019) examined effects of another stimulant, MA, administered orally, on FC of subcortical structures to prefrontal cortex in healthy adults. In this study, MA increased connectivity between putamen and inferior frontal cortex, and between nucleus accumbens (NAcc) and medial prefrontal cortex, but decreased connectivity of NAcc to subgenual anterior cingulate. Mueller et al. (2014) examined effects of methylphenidate, another stimulant with a slightly different mechanism of action (Volkow et al. 2002; Chiu et al. 2012), on connectivity in selected RSNs identified with ICA. Methylphenidate increased connectivity among several cortical regions, including visual and sensorimotor cortex. Also using methylphenidate and a seed-based connectivity approach, Ramaekers et al. (2013) found that methylphenidate decreased connectivity of many portions of cortex to the NAcc (but did not change thalamus connectivity). Thus, the findings with stimulant drugs are inconsistent, probably due to methodological variations in identifying networks and assessing for connectivity, as well as differences in drugs, dosing, and subject samples. Nevertheless, the findings indicate that stimulant drugs affect connectivity across brain regions in several major neural networks.

This study examined effects of oral MA on connectivity across RSNs using a hypothesis-free approach. The hypothesis-free approach may help to resolve inconsistencies in prior findings that examined networks or regions of a priori interest, allowing for observations of changes in cortico-striatal-thalamo-cortical systems, and in simpler regional networks such as visual or sensorimotor cortex. Without selecting networks on an a priori basis, the hypothesis-free analysis can reveal unexpected drug-induced changes in connectivity. We examined MA effects in an unrestricted manner by first using a data-driven approach to categorizing all cortical and subcortical parts of the brain into RSNs, then for each, contrasting MA with placebo (PL). We also separately examined between-network and within-network connectivity. This approach should allow us to verify robust prior findings and to identify as-yet unknown drug actions on brain function, although at the possible cost of insensitivity to weaker effects (type II error). In secondary analyses, we also examined individual differences in connectivity in relation to participants’ subjective reports of the drug’s effects, including drug liking and stimulant-like effects.

## Materials and Methods

### Subjects

Healthy subjects 18 to 35 years of age (*n* = 22, 12 women) were recruited through flyers and online advertisement. They were screened with a physical examination, electrocardiogram, self-reported health and drug-use history, and an abbreviated Structured Clinical Interview with the DSM-5 criteria (APA 2013). Inclusion criteria were fluency in English, right-handedness, a high school education, body mass index ranging from 19 to 26 kg/m^2^, and some lifetime use of a drug for nonmedical purposes. Exclusion criteria were a history of psychosis, severe panic or posttraumatic stress disorders, a substance use disorder within the last year (excluding nicotine), being pregnant or nursing, or use of regular medication (excluding birth control). Twenty-two adults completed the 2 sessions (Supplementary Table S1). Subjects were required to refrain from drug use for 48 h before and 24 h after each session and to refrain from cannabis use for 7 days before and 24 h after each session. Consumption of normal amounts of caffeine and nicotine were permitted up to 2 h before the session. Subjects were instructed to get a normal night of rest and to fast the morning of the session. A granola bar was provided at the beginning of each session. Subjects were told they might receive a PL, stimulant, or sedative drug. All subjects provided informed consent before beginning the study. All procedures were approved by the University of Chicago Institutional Review Board.

### Study Design and Procedure

This study used a double-blind, within-subject design consisting of 2 sessions where subjects received either PL or MA (20 mg oral) in counterbalanced order. The 2 4-h study sessions were scheduled from 9 AM to 1 PM, separated by at least 4 days. Before each session, subjects provided a urine sample to test for recent substance use (ToxCup, Branan Medical Corporation, Irvine, CA) and provided a breath sample (AlcoSensorIII, Intoximeters, St. Louis, MO) to determine breath alcohol content. Positive results resulted in rescheduling. Women were screened for pregnancy (AimStickPBD, hCG professional, Craig Medical Distribution, Vista CA). Naturally cycling women were tested only during the follicular phase of their menstrual cycle. Subjects completed predrug baseline subjective and cardiovascular measures, and at 9:30 AM, they consumed a syrup containing either PL or 20 mg of MA. Subjective and cardiovascular measures were collected 25 min after drug administration and subjects were then escorted to the imaging center for the 45-min MRI scan. After the scan, subjects completed further subjective and cardiovascular measures. After completing both sessions, subjects were told which drug they received and compensated.

### Drug

MA tablets (5 mg, total dose 20 mg; Desoxyn, Lundbeck) were crushed and mixed with 10 mL of combined Ora-Plus and Ora-Sweet syrups (Paddock Laboratories, Minneapolis, MN). This dose and mode of administration reliably produces subjective, cardiovascular, and behavioral effects that peak 30–70 min after drug administration (Mayo et al. 2013; Mayo and de Wit 2015). PL consisted of 10 mL of equal parts Ora-Plus and Ora-Sweet. Syrups were administered in 1 oz plastic cups.

### Subjective and Cardiovascular Measure

Subjects completed the Addiction Research Center Inventory (ARCI; Haertzen 1966) before and 15, 30, 75, 115, and 200 min after drug administration. To provide a single sensitive index of amphetamine-like effects, we examined responses to the Amphetamine (ARCI A; “stimulant-like”) scale. Heart rate (HR) and blood pressure (BP) were monitored (Omron, Lake Forest, IL) at the same intervals as the subjective measures. Mean arterial pressure (MAP) was calculated using the following formula:

**Figure:**
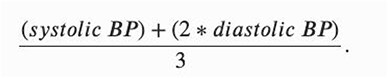


### Image Acquisition and Preprocessing

Resting state data were collected as part of a larger study that also collected task-related data, reported elsewhere (Van Hedger et al. 2019). Scans were collected using a Philips Achieva 3.0 T scanner with a 32-channel head coil. The resting state scan was a gradient-echo echo-planar imaging sequence with the following acquisition parameters: TR = 3000 ms; TE = 30 ms; 46 3-mm thick axial slices aligned to the bicommissural line, 0.30 mm slice gap; 216 × 216 mm field-of-view (2.70 mm^3^ voxels); flip angle = 90°. Four initial volumes were acquired and discarded by the scanner computer to allow for T1 equilibration effects. After that, 124 volumes were acquired. During the scan, subjects viewed a white fixation cross on a black background. In each scanning session, a high resolution *T*_1_-weighted image was acquired for coregistration and normalization. Motion was minimized with foam packing around the head.

Resting state scans were processed using the CONN toolbox (Whitfield-Gabrieli and Nieto-Castanon 2012). Functional data were realigned using SPM12’s realign and unwarp procedure (Andersson 2001), where all scans were coregistered and resampled to the first scan of the session using b-spline interpolation. Slice-timing was corrected using SPM12’s slice-timing correction procedure (Henson et al. 1999). Volumes were identified as motion outliers using CONN’s artifact detection tool, with a subject motion threshold of 0.5 mm and a global signal z-value threshold of 3 standard deviations, following CONN’s “conservative” settings. Subjects were removed from the analysis if mean composite motion was > 0.5 mm and/or they had > 50% of volumes exceeding 0.5 mm composite motion for either scan. Functional and anatomical data were normalized into standard Montreal Neurological Institute space and segmented into gray matter, white matter, and cerebrospinal fluid (CSF) tissue classes using SPM12’s unified segmentation and normalization procedures (Ashburner and Friston 2005). Denoising (per CompCor, implemented in CONN) included regression of white matter and CSF signals, scrubbed volumes, motion +first-order derivatives, a linear and second-order polynomial drift term and application of a bandpass filter from 0.008 to 0.9 Hz (Behzadi et al. 2007). Functional smoothing was applied using a 6 mm full-width at half-maximum Gaussian kernel.

### Drug Effect Analyses

#### Nonimaging Drug Effects

Repeated-measures ANOVA was used to examine subjective and cardiovascular effects of MA and PL. Time (in session) and drug (MA-PL) were treated as within-subject factors.

#### RSN Identification and Within-Network Connectivity Analysis

To identify how MA alters FC within and between functionally distinct RSNs, we first identified RSNs using a meta group-ICA (gICA) of the PL scans. For this, 10 gICA analyses were performed on the PL scans with randomized order of scan input, as it has been shown that subject input order can affect gICA outputs (Wisner et al. 2013). The number of components (RSNs) for each gICA was estimated using the Laplacian approximation to the Bayesian evidence of the model order (Beckmann et al. 2004), maximizing the differentiation of RSNs from one another. To identify networks robustly present across the 10 gICAs, each network was correlated against all other networks using FSL’s *fslcc* command. Any set of 10 components where all components correlated with one another > 0.7 and were from different gICAs were then averaged together to produce the final RSNs for within- and between-network drug versus PL comparisons.

For within-network connectivity analysis of drug effects, we used dual regression (Beckmann et al. 2009). Dual regression calculates for each subject’s MA and PL resting state scans, spatial maps, and associated time courses corresponding to each of the gICA-derived components (e.g., each RSN). A spatial regression is performed first, regressing the components derived from the gICA onto each subject’s functional data. This results in a time x component set of beta weights that describe the temporal dynamics of each component within each subject’s resting state scan. A temporal regression is then performed, regressing the temporal dynamics onto each subject’s resting state scans, resulting in a set of spatial maps that quantify each voxel’s fit with each component (Smith et al. 2014). Data were normalized to unit variance. These spatial maps were then input into 1 within-group comparison for each RSN using the general linear model (GLM) to compare MA to PL. Clusters of activation were estimated using threshold-free cluster enhancement (Smith and Nichols 2009). Significance was assessed via a permutation-based, Bonferroni-corrected approach (5000 permutations per GLM, Nichols and Holmes 2002; familywise alpha = 0.05 achieved by evaluated clusters from any GLM as significant at *P* > 0.001).

**Figure 1:**
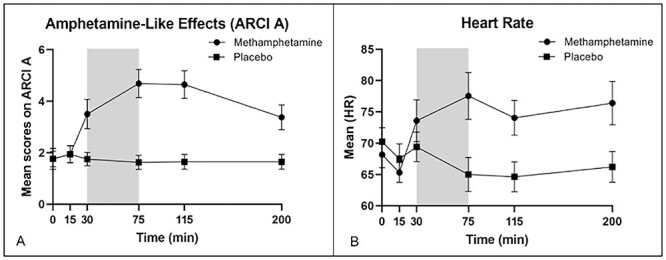
Subjective and cardiovascular responses to MA. Mean (and standard error of mean [SEM]) values at each time point in minutes after drug administration, on ARCI Amphetamine scale (*A*) and heart rate (*B*). The gray bars indicate when the MRI scan occurred. Square symbols refer to MA and circles to placebo. MA significantly increased both heart rate and subjective ratings as measured by the ARCI A scale.

#### Between-Network Connectivity Analysis

To identify how MA alters FC between the RSNs, we used the CONN ROI-to-ROI connectivity (RRC) tool. RSNs identified in the meta-gICA procedure were entered as ROIs in CONN. Then, for both MA and PL sessions, RRC correlation matrices were computed, representing the level of FC between each pair of RSNs and expressed as Fisher-transformed bivariate correlation coefficients (Whitfield-Gabrieli and Nieto-Castanon 2012). A standard second-level GLM analysis of RRC matrices was conducted to compare MA to PL. Significance was assessed via the nonparametric network-level inference approach (Zelesky et al. 2010), with a bivariate connection threshold of *P* < 0.01 and a cluster threshold of *P* < 0.05 (network-level family-wise error corrected).

#### Relationship between FC and Subjective Response to MA

We examined the association of MA effects on within-network FC in relation to its effects on subjective ratings of drug effects by correlating change scores of each. For within-network FC change scores, we extracted mean component fit (mean beta-weight) from each significant cluster of voxels from the group analysis for each subject’s MA and PL FC map. Then we computed difference scores of these means (MA-PL). For between-network change scores, for each significantly altered network-to-network connectivity, we extracted subjects’ individual correlations between those network pairings for PL and then for MA, and subtracted them (MA-PL), yielding change scores for each subject representing changed connectivity between the network pair that had shown significant drug effects at the group level. For subjective ratings change scores, peak change scores (PCS) were calculated for ARCI “A” from each MA and PL session and subtracted (MA-PL). Pearson’s correlation coefficients were calculated between within-network FC change scores and subjective response change scores, and for between-network FC change scores and subjective response change scores. Results were evaluated as significant following a Bonferroni correction.

**Figure 2:**
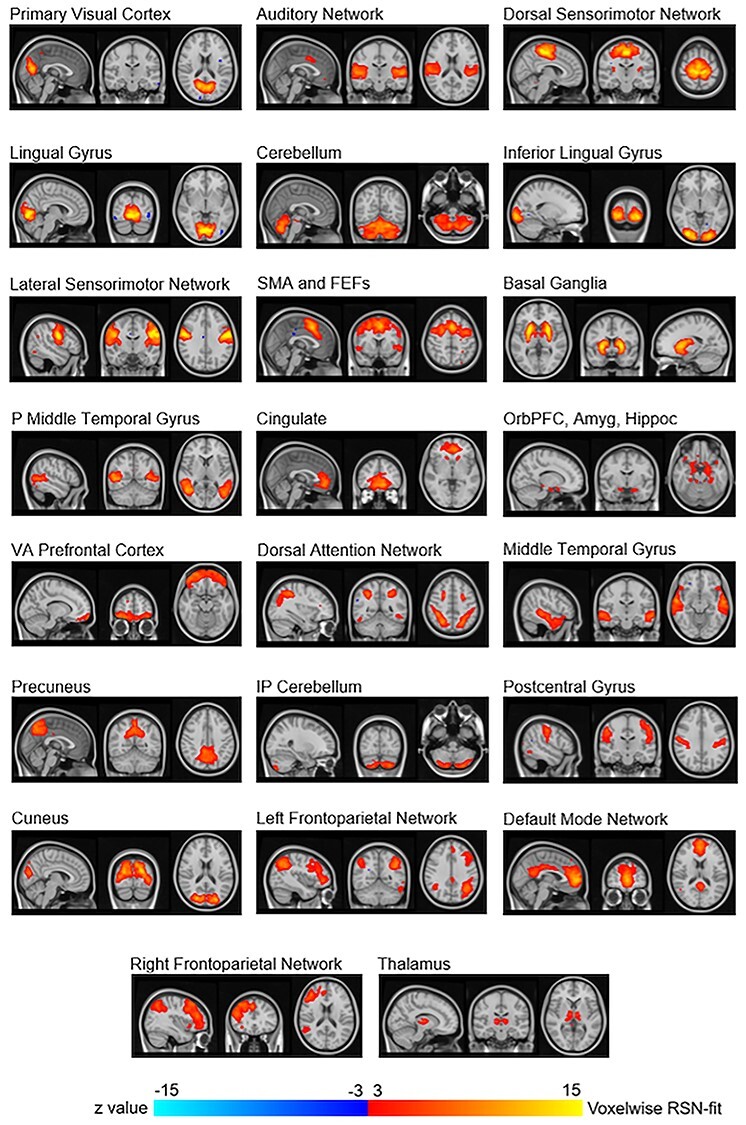
Resting state networks that were visually identified based on placebo session data. These 23 networks were averaged components from the 10 gICAs. SMA and FEFs: Supplementary motor area and frontal eye fields; P Middle Temporal Gyrus: Posterior middle temporal gyrus; OrbPFC, Amyg, Hippoc: Orbitofrontal prefrontal cortex, amygdala, and hippocampus; VA Prefrontal Cortex: Ventral anterior prefrontal cortex.

## Results

### Subjective Drug Effects and Motion

MA produced its expected effects on both subjective and cardiovascular measures (Mayo and de Wit 2015; Van Hedger et al. 2019). The drug increased ARCI A (drug x time; *F*_5,65_ = 8.77, *P* < 0.001; Fig. 1), HR (drug x time; *F*_5,85_ = 13.71, *P* < 0.001), and MAP (drug x time; *F*_5,85_ = 5.84, *P* < 0.001). No subjects met exclusion criteria for excessive motion (mean excluded volumes = 13%). There was no difference between MA and PL on residual motion (MA mean = 0.15 ± 0.01; PL mean = 0.19 ± 0.02; t = 1.75, *P* = 0.09).

### RSN Identification

The 10 gICAs yielded outputs ranging from 31 to 43 components (mean = 35.3, standard deviation [SD] = 3.1). Of the 353 total components across the 10 gICAs, 30 “primary components” had a spatial correlation of at least 0.7 with 9 other components (Supplementary Table S2 shows correlation values for nonnoise components). About 8 of those 30 components were visually identified as artifact, leaving 22 identified as consistent with functional RSNs. Of the 10 gICAs conducted, only 4 had thalamus components correlating among each other at > 0.7. We averaged these 4 components together to produce a thalamus component to allow for assessment of this network as well, resulting in 23 total RSNs for the within- and between-network analyses (Fig. 2). Some RSNs were spatially continuous voxel groups overlaying brain regions such as visual cortex portions. Other RSNs were multinodal such as frontoparietal dorsal attention and default mode.

### Within-Network Connectivity Drug Effects

MA did not significantly alter within-network FC across the brain at the Bonferroni-corrected threshold of *P* < 0.05 (where any cluster had to be at *P* < 0.001; Supplementary Table S3). Results at more liberal thresholds are shown in Supplementary Figure S1 and Supplementary Table S4, and we used these clusters to assess correlation of within-network changes to subjective drug effects.

### Between-Network Connectivity Drug Effects

**Figure 3:**
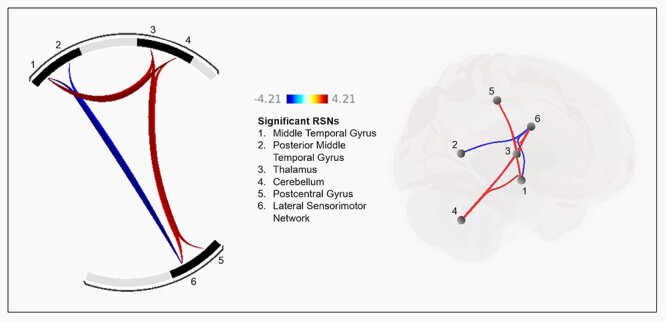
Effect of MA on between-network functional connectivity. The left shows pairs of networks that were significantly increased (red connections) or decreased (blue connections) after MA relative to PL. Numbers correspond to resting state networks listed in the center. The right shows the anatomical location of the 7 significant connections of the resting state networks. Color scale represents *t*-values.

MA significantly altered connectivity between 7 pairs of RSNs (Table 1, Fig. 3). Two pairs showed decreased connectivity following MA administration and were lateral sensorimotor network and middle temporal gyrus, and lateral sensorimotor network and posterior middle temporal gyrus. The remaining 5 pairs showed increased connectivity following MA administration and were cerebellum and middle temporal gyrus, cerebellum and lateral sensorimotor network, thalamus and postcentral gyrus, thalamus and middle temporal gyrus, and thalamus and lateral sensorimotor network.

**Table 1:** Significant between-network increases and decreases in connectivity after MA compared to PL

PL > MA connectivity	t	*P*
Lateral sensorimotor network and middle temporal gyrus	--3.97	0.0007
Lateral sensorimotor network and posterior middle temporal gyrus	--3.03	0.0064
**MA > PL connectivity**		
Cerebellum and middle temporal gyrus	4.21	0.0004
Thalamus and postcentral gyrus	3.64	0.0015
Thalamus and middle temporal gyrus	3.42	0.0025
Cerebellum and lateral sensorimotor network	3.4	0.0027
Thalamus and lateral sensorimotor network	3.4	0.0027

Note: These connections correspond with the data presented in Figure 3, in which increases in connectivity after MA are presented in red and decreases in connectivity are in blue.

### FC and Subjective Drug Effects

No significant correlations were observed between subjective responses to MA and within- or between-network FC change (Supplementary Table S3).

## Discussion

In this study, we sought to identify effects of MA on within- and between-network connectivity of resting state brain networks in healthy individuals, using a hypothesis-free approach. We applied this approach both to identify brain networks and to assess MA-induced changes. MA did not significantly alter within-network connectivity. Between networks, MA had mixed effects, increasing FC between some networks and decreasing it between others. MA increased connectivity between the thalamus and 3 cortical networks, and between cerebellum and two of the 3 cortical networks that increased connectivity to thalamus. In contrast, MA decreased connectivity between 2 pairs of cortical networks—those that had increased connectivity to thalamus. Finally, although MA produced its expected subjective effects, these were not significantly related to any changes in FC.

In contrast to previous studies that defined networks based on prior knowledge of drug actions, we used an optimized ICA-solution approach that maximized independence of RSNs from one another and from noise. While prior studies have examined effects of stimulant drugs using ICA to identify RSNs, none have used ICA to identify optimally distinct RSNs and then assessed each RSN for drug effects. This broad, data-driven approach was thought to be potentially informative because MA has such diverse behavioral and physiological effects, each of that could be mediated by different underlying processes (e.g., response to reward, Knutson et al. 2004; Bernacer et al. 2013; treatment response in ADHD, Pereira-Sanchez et al 2020; response to visual stimuli, Van Hedger et al. 2019).

### Within-Network Connectivity

We did not find that MA significantly changed within-network connectivity. This finding contrasts with Schrantee et al. (2016) who found that intravenous d-amphetamine decreased connectivity between anterior cingulate and a large scale cortico-striatal-thalamic network. Differences may be due to drugs (MA vs. d-amphetamine), dosing, routes of administration or subject samples, or due to differences in how the networks were defined. Of note, our nominal findings (Supplementary Fig. S1) were comparable to those reported in Schrantee et al., including their primary and their exploratory findings in other networks, in that they were all in the direction of decreased connectivity. Our approach to testing all networks for drug effects necessitated higher significance thresholds per network, but both studies show similar trends, suggesting that amphetamine reduces coherence of neural activity within networks.

### Between-Network Connectivity

MA increased FC between several networks: specifically, with thalamus to sensorimotor or middle temporal cortex and with cerebellum to similar cortical networks. The increase in FC of thalamus to the cortical networks is in contrast to a study using a different stimulant, methylphenidate, which found no change in connectivity between the thalamus and other cortical regions (Ramaekers et al. 2013). However, our finding is in line with hypotheses that stimulants affect normal connectivity of the cortico-striato-thalamo-cortical networks, networks that are known to be involved in behavioral effects of stimulants (Jentsch and Taylor 1999; Goldstein and Volkow 2011). The MA-induced increases in connectivity of thalamus to sensorimotor RSNs (as both postcentral and lateral sensorimotor networks are) may be related to the drug’s effects on increased motor activity.

The spatial location of the cortical networks with increased connectivity shows some overlap to exploratory findings of the salience network reported in Schrantee et al. (2016), where clusters of decreased connectivity within that network fall along similar sensorimotor and temporal lobe regions to our sensorimotor and temporal networks showing increased connectivity to thalamus. In short, both studies suggest amphetamine alters connectivity of the sensorimotor and middle temporal lobe, although the nature of the alterations is inconsistent.

In this study, MA increased connectivity between cerebellum and some of the same cortical networks with increased connectivity to thalamus. As this appears to be the first report to include a cerebellar network to assess amphetamine effects on resting state connectivity, this finding needs further study. However, MA also decreased FC between the same cortical networks that had increased connectivity to thalamus and cerebellum, suggesting a systems-level effect. Typically, adjacent cortical networks, such as these sensorimotor and temporal networks, are more synchronous with one another than more distal regions. The observation that MA increased synchronization with subcortical structures (thalamus and cerebellum) but decreased synchronization with adjacent regions is intriguing. MA is thought to act primarily via dopaminergic projections from the substantia nigra (Volkow and Morales 2015), but it is likely to affect broad regions including the thalamus. Why the effect was apparent only for limited thalamic-cortical associations is unclear.

### No Correlations with Subjective Drug Effects

Finally, we did not observe any associations between subjective ratings of drug effects and FC changes. This may be due to the lack of variance in responses to the drug, relatively small sample, or low dose. Alternatively, subjective reports of the drug experience may not be related to the specific brain RSN connectivity changes studied here. Stimulant drugs have multiple behavioral and physiological effects, which may be mediated by different neural processes (Sulzer et al. 2005). That said, Weafer et al. (2020) examined relationships between subjective responses to MA and drug-induced changes in FC in a seed-based, frontally focused analysis and found that individuals who exhibited greater drug-induced increase in FC between left inferior frontal gyrus and putamen reported less euphoria and stimulation. This suggests that a more focused, hypothesis-driven analytic approach may be more likely to detect associations between drug effects on brain connectivity and subjective response.

### Limitations and Future Directions

This study has a number of limitations. First, the sample was relatively small and homogeneous, consisting of healthy young adults with few psychiatric symptoms and minimal prior drug use experience. It will be important to extend these findings to a broader population, including those who might be at risk for substance use disorders. Furthermore, influence of residual motion cannot be entirely ruled out as influencing results, further calling for replication. Second, our FC data portrayed neural function changes only at rest. It will be important for future studies to assess connectivity changes during task performance, as this might reveal a different pattern of brain activity that could have further implications for substance use disorders. Finally, we did not include a perfusion measure to assess global blood flow effects. However, this concern is somewhat mitigated by the finding that the drug affected specific neural systems and not others.

The present findings add to our understanding of how stimulant drugs affect brain function. Previous studies on the effects of stimulants on resting state FC have yielded mixed results (Ramaekers et al. 2013; Mueller et al. 2014; Konova et al. 2015; Schrantee et al. 2016; Gorka et al. 2020; Weafer et al. 2020), perhaps because they targeted selected brain regions of a priori interest. Here, we used a hypothesis-free independent component analysis-based approach to examine effects of MA on FC. The drug did not significantly alter connectivity within networks, but it altered connectivity between certain networks. MA increased connectivity from both thalamus and cerebellum to sensorimotor and middle temporal gyrus, but decreased connectivity between these cortical networks. These findings extend our understanding of the actions of MA on brain function, which may help to optimize their use in clinical settings and to understand the development of substance use disorders. Future studies with larger and more heterogeneous samples will shed light on relationships between these neural actions and the behavioral, cognitive, and motivational effects of stimulant drugs.

## Funding

National Institutes of Health (grant DA02812). KVH is supported by a BrainsCAN Tier 1 Postdoctoral Fellowship. JW is supported by National Institutes of Health (grants R01AA028503 and K01AA024519). The authors acknowledge the support of the University of Chicago Magnetic Resonance Imaging Research Center funded by S10OD018448, and the University of Chicago Research Computing Center.

## Notes

*Conflict of Interest*: None declared

## Supplementary Material

FigS1_color_tgab063

Malina_supplemental_tgab063
