# Supplementary figures and images for: Effects of Methamphetamine on Within- and Between-Network Connectivity in Healthy Adults
Source: Cereb Cortex Commun. 2021 Oct 29;2(4):tgab063. doi: 10.1093/texcom/tgab063 (PMC8633740; doi:10.1093/texcom/tgab063)

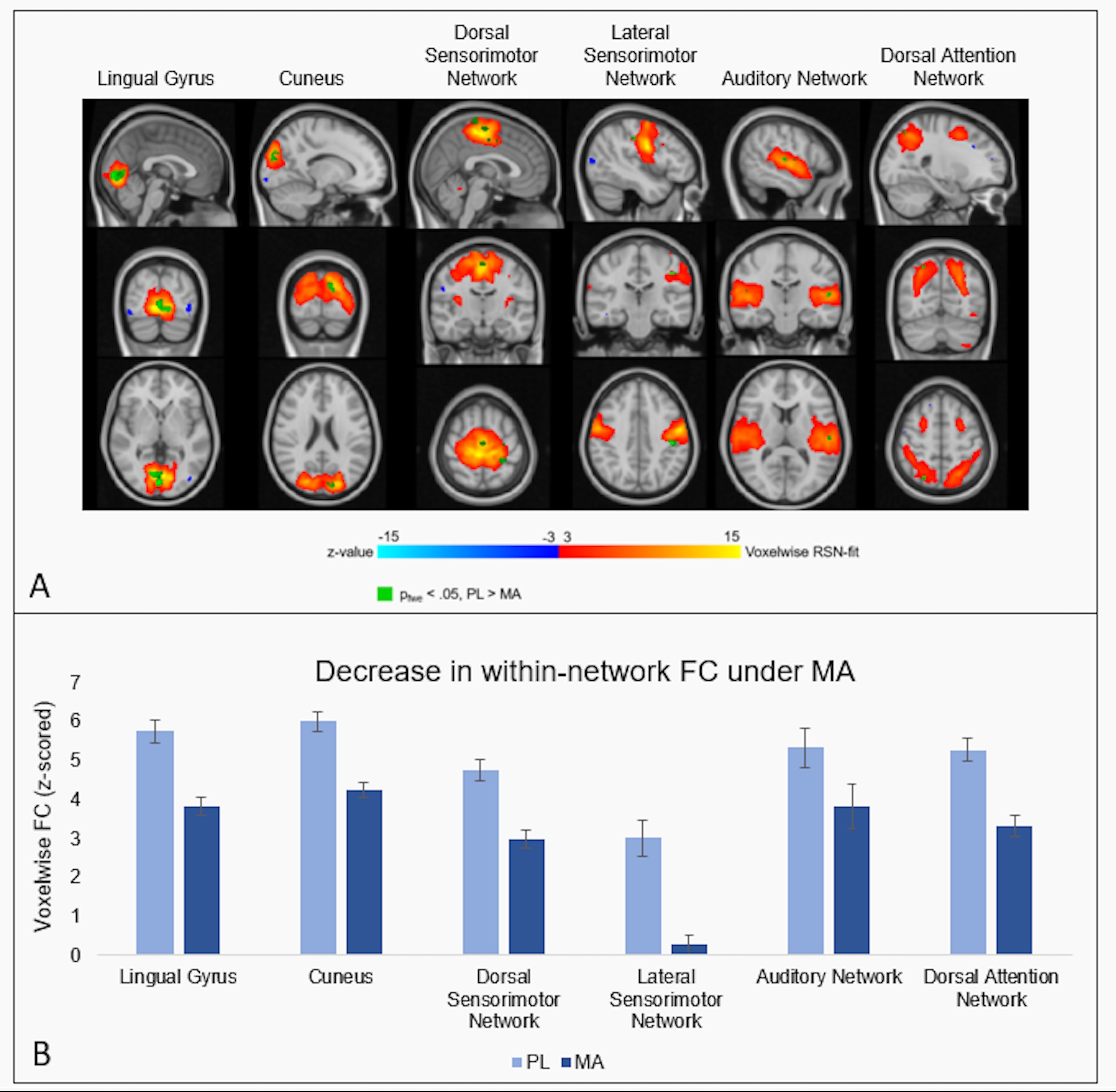

Supplement: FigS1_color_tgab063 [file figs1_color_tgab063.jpeg]
