# Supplementary material for: Effects of Methamphetamine on Within- and Between-Network Connectivity in Healthy Adults
Source: Cereb Cortex Commun. 2021 Oct 29;2(4):tgab063. doi: 10.1093/texcom/tgab063 (PMC8633740; doi:10.1093/texcom/tgab063)

**Supplementary Materials**

**Within-network functional connectivity at liberal threshold**

The effects of MA on within-network functional connectivity did not reach statistical significance at the p levels corrected for multiple comparisons. However, MA decreased connectivity relative to PL within 6 RSNs (Table S4, Figure S1) at an uncorrected p value < 0.05. MA decreased within-network connectivity compared to PL in portions of two visual cortex networks located in lingual and cuneus gyri, two sensorimotor networks, auditory network and in parietal portions of the dorsal attention network.

Table S1. Demographic characteristics and nonmedical drug use for the 22 participants. *Means for frequency of drug use are calculated based on subjects who report any recent use, indicated as n.

|  | **N or Mean (SD)** |
| --- | --- |
| Sex (M, F) | 10, 12 |
| Age | 27.3 (3.4) |
| Education (years) | 15.8 (1.2) |
| Race |  |
| Caucasian | 13 |
| African American | 5 |
| Asian | 2 |
| Other | 2 |
| Current drug use  Caffeinated drinks (per day*) | 2.2 (0.2), n = 18 |
| Cigarettes (per day) | 6.7 (2.3), n = 3 |
| Alcoholic drinks (per week) | 8.2 (0.5), n = 15 |
| Lifetime drug use (N ever used non-medically)  Cannabis | 18 |
| Stimulants  Opiates  Hall  MDMA  Sedatives | 6  4  4  5  2 |

Table S2. Spatially-correlated components identified from the placebo session. To produce our resting state networks used for assessment of drug effects, we conducted 10 different gICAs and then combined highly spatially correlated components across all 10 gICAs. The 10 gICAs resulted in a total of 353 components. For this table, we assigned each component a unique identifying number 1-353 (for example, the first of the 10 gICAs yielded 43 components and were assigned 1-43; the second gICA yielded 35 components and were assigned numbers 44-79, etc). “Principal components” are those that had the highest (r >0.7) spatial correlations with components among the other 9 gICAs. Each set of highly correlated components were spatially averaged to serve as the networks assessed for drug effects. Only brain resting state networks are shown (as opposed to noise components). Rows show component number, correlation value (r) with the “principal component” (which is just arbitrarily one of the two components with the very highest correlation), and are ordered within each row highest to lowest correlation.

|  |  | Component, r value with principal component | | | | | | | | |  |
| --- | --- | --- | --- | --- | --- | --- | --- | --- | --- | --- | --- |
|  | Principal  Component | 2 | 3 | 4 | 5 | 6 | 7 | 8 | 9 | 10 | Mean  r-value |
| Primary Visual Cortex | 1 | 288,0.92 | 79, 0.91 | 214, 0.88 | 112, 0.86 | 180, 0.84 | 250, 0.84 | 319, 0.82 | 44, 0.82 | 145, 0.82 | 0.86 |
| Auditory Network | 2 | 320,0.9 | 45, 0.9 | 113, 0.84 | 80, 0.83 | 181, 0.81 | 146, 0.81 | 215, 0.8 | 291, 0.77 | 251, 0.76 | 0.82 |
| Dorsal Sensorimotor Network | 5 | 252,0.88 | 46, 0.88 | 322, 0.87 | 147, 0.86 | 216, 0.76 | 183, 0.74 | 81, 0.72 | 114, 0.72 | 290, 0.7 | 0.83 |
| Lingual Gyrus | 7 | 220,0.9 | 293, 0.9 | 83, 0.84 | 182, 0.84 | 117, 0.84 | 47, 0.84 | 253, 0.82 | 321, 0.82 | 148, 0.74 | 0.83 |
| Cerebellum | 13 | 265,0.87 | 59, 0.84 | 229, 0.84 | 334, 0.82 | 195, 0.79 | 161, 0.79 | 128, 0.75 | 304, 0.74 | 94, 0.74 | 0.8 |
| Inferior Lingual Gyrus | 14 | 259,0.91 | 223, 0.9 | 189, 0.78 | 88, 0.77 | 121, 0.77 | 54, 0.76 | 152, 0.74 | 327, 0.73 | 298, 0.71 | 0.78 |
| Lateral Sensorimotor Network | 17 | 264,0.88 | 228, 0.87 | 157, 0.85 | 194, 0.83 | 58, 0.81 | 127, 0.81 | 333, 0.79 | 92, 0.77 | 301, 0.75 | 0.81 |
| SMA and FEFs | 18 | 268,0.9 | 96, 0.88 | 163, 0.87 | 129, 0.83 | 233, 0.83 | 338, 0.81 | 307, 0.8 | 61, 0.76 | 202, 0.74 | 0.85 |
| Basal Ganglia | 27 | 275,0.84 | 239, 0.78 | 342, 0.76 | 66, 0.75 | 170, 0.75 | 136, 0.73 | 204, 0.73 | 102, 0.72 | 312, 0.71 | 0.78 |
| BPM Temporal Gyrus | 28 | 272,0.86 | 237, 0.85 | 165, 0.85 | 63, 0.84 | 340, 0.84 | 99, 0.81 | 134, 0.79 | 199, 0.77 | 306, 0.75 | 0.82 |
| Cingulate | 33 | 104,0.92 | 249, 0.91 | 286, 0.88 | 203, 0.86 | 317, 0.83 | 135, 0.81 | 179, 0.79 | 78, 0.75 | 353, 0.73 | 0.83 |
| OrbPFC, Amyg, Hippoc | 34 | 279,0.89 | 244, 0.85 | 347, 0.85 | 139, 0.78 | 208, 0.77 | 175, 0.77 | 73, 0.75 | 107, 0.73 | 315, 0.73 | 0.79 |
| VA Prefrontal Cortex | 39 | 283,0.91 | 248, 0.88 | 76, 0.88 | 177, 0.84 | 350, 0.83 | 213, 0.83 | 110, 0.82 | 143, 0.81 | 318, 0.79 | 0.84 |
| Dorsal Attention Network | 48 | 219,0.88 | 185, 0.87 | 118, 0.86 | 85, 0.86 | 150, 0.86 | 258, 0.85 | 6, 0.85 | 294, 0.84 | 330, 0.79 | 0.85 |
| BM Temporal Gyrus | 51 | 184,0.84 | 153, 0.84 | 262, 0.83 | 116, 0.83 | 84, 0.83 | 217, 0.81 | 292, 0.8 | 331, 0.76 | 10, 0.74 | 0.83 |
| Precuneus | 60 | 193,0.89 | 160, 0.88 | 231, 0.88 | 267, 0.88 | 126, 0.86 | 93, 0.85 | 336, 0.85 | 300, 0.84 | 20, 0.84 | 0.86 |
| IP Cerebellum | 72 | 345,0.87 | 173, 0.84 | 316, 0.84 | 210, 0.84 | 141, 0.84 | 243, 0.84 | 108, 0.83 | 280, 0.83 | 35, 0.82 | 0.85 |
| Postcentral Gyrus | 124 | 197,0.9 | 238, 0.89 | 273, 0.89 | 332, 0.87 | 169, 0.86 | 65, 0.83 | 309, 0.83 | 19, 0.81 | 101, 0.81 | 0.84 |
| Cuneus | 165 | 63,0.88 | 272, 0.87 | 237, 0.86 | 340, 0.83 | 199, 0.83 | 99, 0.83 | 134, 0.82 | 306, 0.81 | 28, 0.8 | 0.83 |
| Left Frontoparietal Network | 232 | 255,0.81 | 149, 0.81 | 50, 0.8 | 325, 0.79 | 86, 0.78 | 119, 0.76 | 186, 0.75 | 31, 0.7 | 296, 0.7 | 0.81 |
| Default Mode Network | 260 | 227, 0.86 | 12, 0.85 | 326, 0.84 | 89, 0.84 | 133, 0.83 | 156, 0.82 | 196, 0.82 | 49, 0.81 | 295, 0.81 | 0.83 |
| Right Frontoparietal Network | 289 | 82, 0.9 | 115, 0.88 | 3, 0.88 | 218, 0.87 | 254, 0.86 | 187, 0.84 | 154, 0.83 | 52, 0.83 | 324, 0.83 | 0.86 |
| Thalamus | 35 | 53,0.89 | 257, 0.74 | 224, 0.7 |  |  |  |  |  |  | 0.78 |

Table S3 Minimum p-value for drug effects (MA vs PL) on within-network connectivity for each Resting State Network. P-Values shown represent the strongest effects observed for both increases and decreases in within-network FC under MA compared to placebo. None of these reached Bonferroni-corrected significance. Full RSN names can be found in Figure 2.

|  | Minimum  p-value |  |
| --- | --- | --- |
| **Network** | **PL > MA** | **PL < MA** |
| Primary Visual Cortex | 0.061 | 0.375 |
| Auditory Network | 0.019 | 0.287 |
| Dorsal Sensorimotor Network | 0.015 | 0.341 |
| Lingual Gyrus | 0.005 | 0.205 |
| Cerebellum | 0.065 | 0.289 |
| Inferior Lingual Gyrus | 0.391 | 0.395 |
| Lateral Sensorimotor Network | 0.005 | 0.372 |
| SMA and FEFs | 0.110 | 0.427 |
| Basal Ganglia | 0.391 | 0.449 |
| P Middle Temporal Gyrus | 0.361 | 0.246 |
| Cingulate | 0.185 | 0.205 |
| OrbPFC, Amyg, Hippoc | 0.296 | 0.585 |
| VA Prefrontal Cortex | 0.264 | 0.379 |
| Dorsal Attention Network | 0.018 | 0.260 |
| Middle Temporal Gyrus | 0.103 | 0.058 |
| Precuneus | 0.651 | 0.102 |
| IP Cerebellum | 0.268 | 0.263 |
| Postcentral Gyrus | 0.361 | 0.246 |
| Cuneus | 0.015 | 0.525 |
| Left Frontoparietal Network | 0.066 | 0.501 |
| Default Mode Network | 0.298 | 0.336 |
| Right Frontoparietal Network | 0.163 | 0.348 |
| Thalamus | 0.502 | 0.130 |

Table S4. RSNs in which MA significantly decreased within-network FC compared to PL at a more liberal threshold (p<.05). Mean FC difference factor refers to the magnitude of difference between MA and PL FC within the significant clusters. Mean FC difference factor was calculated by dividing the mean z-scored beta-weight across all significant clusters in the RSN in the MA condition by that from the PL condition.

| **Component** | **Region** | **p_fwe_** | **MNI coordinates** | **Cluster Size** | **Mean FC difference factor** |
| --- | --- | --- | --- | --- | --- |
| Lingual Gyrus | R Lingual Gyrus | 0.029 | (8,-78,-20) | 664 | 1.507 |
|  | L Lingual Gyrus | 0.04 | (-20,-72,4) | 69 |  |
|  | L Lingual Gyrus | 0.0444 | (24,-76,12) | 36 |  |
| Cuneus | L Superior Occipital Gyrus | 0.0432 | (-14,90,18) | 113 | 1.417 |
| Dorsal Sensorimotor Network | R Supplementary Motor Area | 0.0436 | (2,18,60) | 188 | 1.604 |
|  | L Superior Parietal Lobule | 0.0488 | (-24,-42,60) | 76 |  |
|  | L Middle Frontal Gyrus | 0.0488 | (024,018,52) | 14 |  |
|  | L Supplementary Motor Area | 0.0457 | (2,-12,46) | 10 |  |
| Lateral Sensorimotor Network | L Inferior Parietal Lobule | 0.0084 | (-42,-26,38 | 25 | 11.46 |
| Auditory Network | L Superior Temporal Gyrus | 0.033 | (-50,-22,-6) | 21 | 1.391 |
|  | R Superior Temporal Gyrus | 0.0496 | (50,-18,12) | 17 |  |
|  | R Superior Temporal Gyrus | 0.0499 | (56,-30,16) | 9 |  |
| Dorsal Attention Network | R Superior Parietal Lobule | 0.0282 | (26,-68,50) | 14 | 1.599 |

Table S5 Correlations between MA (vs PL) effects on network connectivity and subjective effects. Within-network results represent correlations between drug effects on within-network FC (MA minus PL) and drug effects on the ARCI ‘A’ subjective rating scale (MA minus PL). Between-network results represent correlations between drug effects on between-network FC (MA minus PL) and drug effects on the ARCI ‘a’ (MA minus PL). *Significance did not survive multiple comparisons correction.

| Within-network | r | p |
| --- | --- | --- |
| Lingual Gyrus | 0.126 | 0.578 |
| Cuneus | -0.1 | 0.963 |
| Dorsal Sensorimotor Network | -0.19 | 0.398 |
| Lateral Sensorimotor Network | -0.221 | 0.323 |
| Auditory Network | -0.096 | 0.672 |
| Spatial Attention Network | -0.345 | 0.116 |
| Between-Network |  |  |
| Cerebellum and Midtemporal Gyrus | 0.434 | 0.043* |
| Thalamus and Postcentral Gyrus | -0.068 | 0.765 |
| Thalamus and Midtemporal Gyrus | 0.225 | 0.314 |
| Cerebellum to Lateral Sensorimotor Network | -0.078 | 0.731 |
| Thalamus to Lateral Sensorimotor Network | -0.079 | 0.727 |
| Lateral Sensorimotor Network to Midtemporal Gyrus | -0.24 | 0.283 |
| Lateral Sensorimotor Network to Middle Posterior Temporal Gyrus | -0.36 | 0.1 |

Figure S1 Effect of MA on within-network functional connectivity at a liberal threshold. Panel A shows within-network clusters in which MA (p_fwe_ < 0.05) decreased FC (green), relative to PL, within each RSN (red-orange-yellow regions). Z-values represent fit that a given voxel has with the network, where high values (over 4.0) indicate a higher likelihood that the voxel is located within the RSN. X-coordinates of the six sagittal slices are 8, -14, 2, -42, -50 and 26, respectively, from left to right. Panel B shows the mean voxelwise FC within each significant cluster following PL (light blue) and MA (dark blue). Errors bars represent the standard error of the mean.


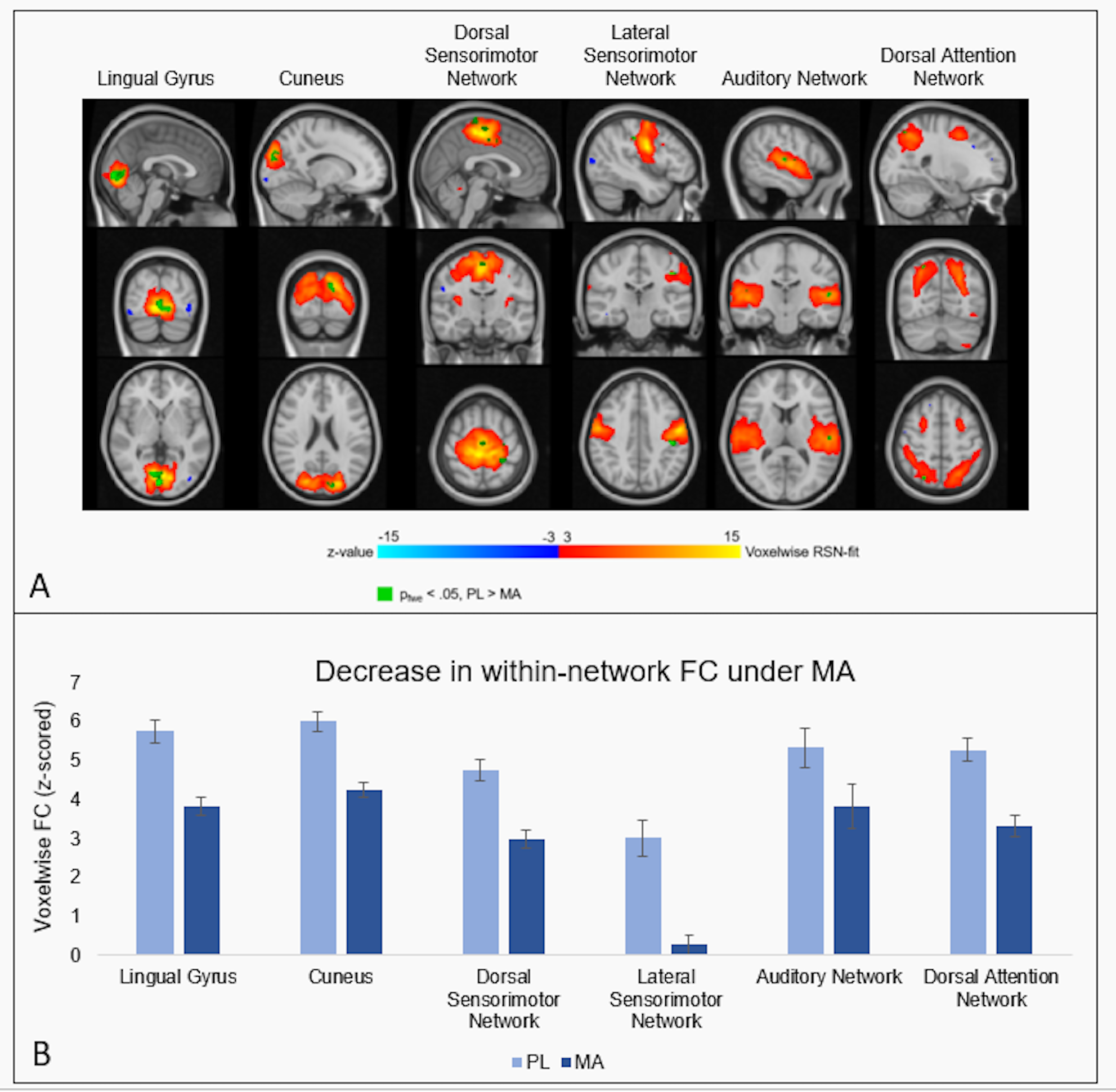

Supplement: Malina_supplemental_tgab063 [file malina_supplemental_tgab063.docx]
